# Gorlin syndrome-induced pluripotent stem cells form medulloblastoma with loss of heterozygosity in *PTCH1*

**DOI:** 10.18632/aging.103258

**Published:** 2020-05-21

**Authors:** Yu Ikemoto, Toshiyuki Miyashita, Michiyo Nasu, Hiromi Hatsuse, Kazuhiro Kajiwara, Katsunori Fujii, Toshino Motojima, Ibuki Kokido, Masashi Toyoda, Akihiro Umezawa

**Affiliations:** 1 Department of Reproductive Biology, National Center for Child Health and Development, Tokyo 157-8535, Japan; 2 Department of Molecular Genetics, Kitasato University Graduate School of Medical Sciences, Sagamihara 252-0374, Japan; 3 Department of Pediatrics, Chiba University Graduate School of Medicine, Chiba 260-8670, Japan; 4 Research Team for Geriatric Medicine (Vascular Medicine), Tokyo Metropolitan Institute of Gerontology, Tokyo 173-0015, Japan

**Keywords:** Gorlin syndrome, induced pluripotent stem cells, medulloblastoma, PTCH1, heterozygosity

## Abstract

Gorlin syndrome is a rare autosomal dominant hereditary disease with a high incidence of tumors such as basal cell carcinoma and medulloblastoma. Disease-specific induced pluripotent stem cells (iPSCs) and an animal model have been used to analyze disease pathogenesis. In this study, we generated iPSCs derived from fibroblasts of four patients with Gorlin syndrome (Gln-iPSCs) with heterozygous mutations of the *PTCH1* gene. Gln-iPSCs from the four patients developed into medulloblastoma, a manifestation of Gorlin syndrome, in 100% (four out of four), of teratomas after implantation into immunodeficient mice, but none (0/584) of the other iPSC-teratomas did so. One of the medulloblastomas showed loss of heterozygosity in the *PTCH1* gene while the benign teratoma, i.e. the non-medulloblastoma portion, did not, indicating a close clinical correlation between tumorigenesis in Gorlin syndrome patients and Gln-iPSCs.

## INTRODUCTION

Gorlin syndrome, a rare autosomal dominant disorder, is characterized by developmental defects in multiple organs or tissues such as the skin (palmar or plantar pits), nervous system, eyes, endocrine systems and bones (bifid ribs). Gorlin syndrome is also associated with tumorigenesis such as development of basal cell carcinoma (BCC), medulloblastoma or keratocystic odontogenic tumor. Gorlin syndrome is caused by mutations in the *PTCH1* gene, a human homologue of *Drosophila patched*. *PTCH1* is a member of the hedgehog signaling complex which is composed of hedgehog, SMO and GLI proteins. Hedgehog signaling regulates cell growth and development, and thus the disorder of this pathway gives rise to not only developmental anomalies but also diverse tumors such as those seen in Gorlin syndrome [1]. Aberrant activation of hedgehog signaling causes basal cell carcinoma [2, 3] and medulloblastoma [4, 5].

Medulloblastoma occurs at an increased rate in mice with germline mutations in the *ptch1* gene. Although mice with *ptch1* deficiency are informative models for studies of Gorlin syndrome and medulloblastoma development, differences in underlying biology exist between humans and mice. Medulloblastoma shows a similar phenotype and anatomical location from haploinsufficiency of *ptch1* in mice, but does not exhibit loss of heterozygosity [6, 7]. Human disease-specific induced pluripotent stem cells (iPSCs) have been used as a human disease model to complement the animal models [8–10]. Basic schemes for how to utilize patient iPSCs for disease mechanism studies have been well described [11–13]. The power of iPSC technology for pathobiology studies is indeed revolutionary. Once established from any given patient, iPSCs serve as enduring resources for providing various functional cell types which retain all of the genomic information from the original patient. Using this system, we should be able to further investigate how disease-related phenotypes develop ‘in a dish’, or even test whether novel therapeutic approaches can reverse the pathogenic changes [10, 13].

In this study, we focus on Gorlin syndrome, one of the well characterized disorders with mutations in the Hedgehog signaling pathway. We have successfully generated a medulloblastoma model with iPSCs derived from patients with Gorlin syndrome (Gln-iPSCs). Interestingly, Gln-iPSCs with a heterozygous germline mutation of *PTCH1* developed into medulloblastoma with a secondary somatic mutation, i.e. loss of heterozygosity (LOH), in *PTCH1* in vivo. This iPSC model may be useful for screening small molecules as drug candidates for treatment of medulloblastoma and Gorlin syndrome.

## RESULTS

### Generation and characterization of Gln-iPSCs

**Figure 1:**
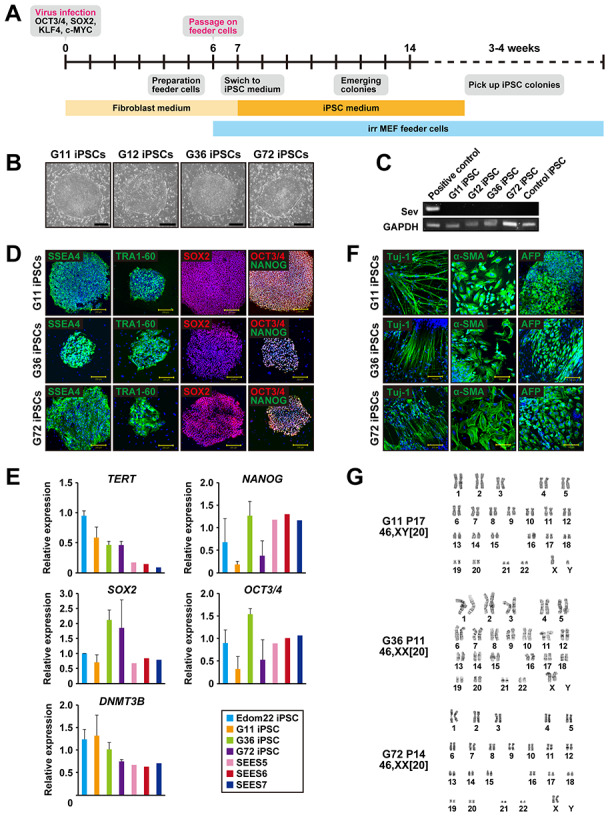
**Generation of iPSCs from fibroblasts of patients with Gorlin syndrome.** (**A**) Protocol for iPSC generation. (**B**) Phase-contrast microphotographs of Gln-iPSCs (G11, G12, G36, G72). (**C**) RT-PCR analysis of the Sendai virus. (**D**) Immunocytochemical analysis of Gln-iPSCs using antibodies to NANOG, OCT4/3, SOX2, SSEA4, and TRA1-60. (**E**) Expression of the endogenous TERT, NANOG, SOX2, OCT4/3, and DNMT3B genes. (**F**) in vitro differentiation of Gln-iPSCs into three germ layers. Immunocytochemical analysis of Gln-iPSCs using antibodies to Tuj-1, α-smooth muscle actin (SMA) and α-fetoprotein (AFP). Expression of the endogenous TERT, NANOG, SOX2, OCT4/3, and DNMT3B genes. (**G**) Karyotypes of Gln-iPSCs at the indicated passage number. Numbers in brackets indicate the number of cells analyzed.

We generated iPSCs from human cells with mutations in the *PTCH1* gene by Sendai virus infection-mediated expression of *OCT4/3*, *SOX2*, *KLF4*, and *c-MYC* (Figure 1A). When the reprogramming factors OCT4/3, SOX2, KLF4 and c-MYC were introduced into 4.0 x 10^5^ cells, iPSCs generated from four patients with Gorlin syndrome were successfully generated and designated as G11-, G12-, G36- and G72-iPSC. Efficiency of iPSC generation was then calculated as “iPSC colonies generated/fibroblasts exposed to virus”. The efficiency of the iPSC colony generation was relatively high, i.e. 0.1% to 1.0%, compared with that of iPSCs (Edom22-iPSCs) generated from healthy individuals. Morphological characteristics of Gln-iPSCs, *i.e.* flat and aggregated colonies, were similar to those of other intact iPSCs and ESCs (Figure 1B). RT-PCR analysis revealed elimination of the Sendai virus (Figure 1C). Immunocytochemical analyses demonstrated expression of the pluripotency-associated markers, *i.e.* SSEA-4, TRA-1-60, SOX2, NANOG, and OCT4/3, which was consistent with the profile observed in hESCs (Figure 1D). The expression profiles of stem cell-associated genes were examined by qualitative RT-PCR to confirm the iPSC-characteristics. Expression of pluripotency-associated genes, such as *SOX2*, *OCT4/3*, *DNMT3B*, *NANOG*, and *TERT,* were detected in all Gln-iPSC clones to a similar extent of those in control human embryonic stem cells and healthy donor-derived iPSCs (Figure 1E). To evaluate whether Gln-iPSCs maintained their pluripotency in vitro, we performed embryoid body (EB) assays. EBs differentiated from Gln-iPSC clones (G11, G36 and G72) expressed markers associated with the three major germ layers: TUJ1 (ectoderm), αSMA (mesoderm), and AFP (endoderm) (Figure 1F). Short tandem repeat (STR) analysis showed clonality between the respective iPSC lines and their parental cells (Table 1). Gln-iPSCs cells showed intact karyotypes (Figure 1G).

**Table 1:** STR analyses of Gorlin iPSCs.

**Locus**	**G11 fibroblasts**		**G11 iPSC**		**G12 fibroblasts**		**G12 iPSC**		**G36 fibroblasts**		**G36 iPSC**		**G72 fibroblasts**		**G72 iPSC**	
D3S1358	15	16	15	16	15	16	15	16	15		15		15	16	15	16
TH01	9		9		6	9	6	9	9		9		9		9	
D21S11	31.2		31.2		30	31.2	30	31.2	29	30	29	30	30		30	
D18S51	15		15		15		15		14	18	14	18	15	16	15	16
Penta_E	14	17	14	17	14	20.2	14	20.2	11	17	11	17	8	14	8	14
D5S818	10	11	10	11	9	11	9	11	10	11	10	11	10	11	10	11
D13S317	8	9	8	9	8		8		8	9	8	9	8	11	8	11
D7S820	10	11	10	11	10	12	10	12	11		11		11		11	
D16S539	10	11	10	11	11	13	11	13	10	12	10	12	9	12	9	12
CSF1PO	11	12	11	12	11	12	11	12	11	12	11	12	9	11	9	11
Penta_D	10		10		9	10	9	10	9	13	9	13	9	10	9	10
AMEL	X	Y	X	Y	X		X		X		X		X		X	
vWA	16	18	16	18	16	18	16	18	16		16		13	19	13	19
D8S1179	13		13		13	14	13	14	10	11	10	11	11	12	11	12
TPOX	8	11	8	11	11		11		8	11	8	11	8		8	
FGA	22	24	22	24	22	24	22	24	21	23	21	23	23	25.2	23	25.2

### Sequence analysis of the *PTCH1* gene

**Figure 2:**
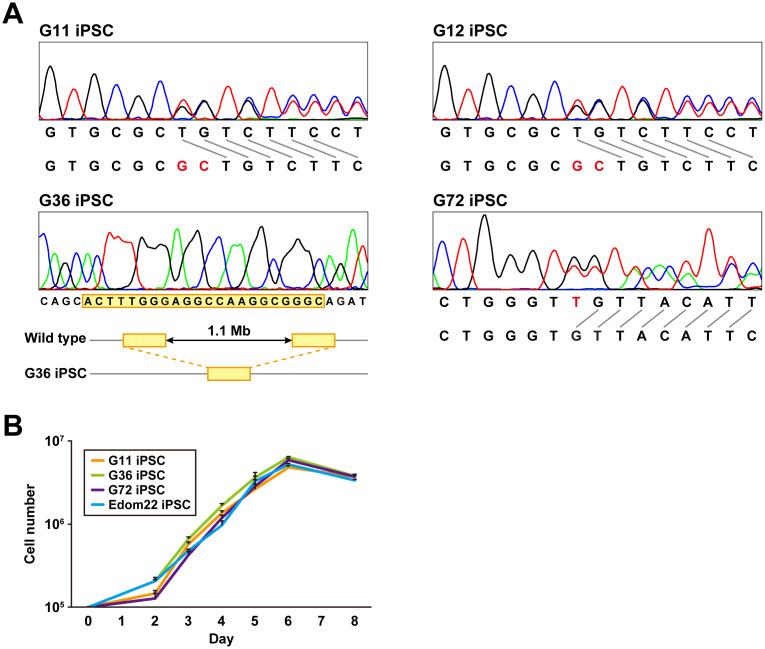
**Genomic analysis and cell proliferation assays of Gln-iPSCs.** (**A**) Sequence analysis of the *PTCH1* gene in Gln-iPSCs. G36 iPSCs contained a ~1.1-Mb deletion with 22-bp overlap (yellow box) which is identical to the parental fibroblast. (**B**) Growth curves of Gln-iPSCs. Cell numbers of Gln-iPSCs (G11, G36, G72) and Edom22-iPSCs (control iPSCs) were counted at the indicated days after cells (1.0 x 10^5^ cells/dish) were seeded on vitronectin-coated 6-well plates.

*PTCH1* mutations of iPSC lines established from four different patients were determined by the direct sequencing method (Figure 2A and Table 2). G12-iPSCs and G11-iPSCs were derived from cells of a mother and son, respectively, and had the same heterozygous mutation, c.3130_3131dupGC, in exon 18, resulting in the frameshift. G36-iPSCs had a heterozygous deletion of the whole *PTCH1* gene. G72 carried a mosaic genotype, wt/c.272delC and wt/c.274delT (Table 2). G72 iPSC clones carrying each of these mutations were established. All mutations identified in iPSCs were identical to those in their parental fibroblasts.

**Table 2:** Phenotypes and genotypes of the patients.

**Patient**	Age/Sex^*1^	**Type of mutation**	**Nucleotide change**	**Amino acid change**	**Symptoms**
G11	14/M	Frameshift	c.3130_3131dupGC	p.V1045LfsX23	Macrocephaly, mental retardation, polydactyly of right lower extremity, palmar pits, rib anomaly
G12	42/F	Frameshift	c.3130_3131dupGC	p.V1045LfsX23	Palmar pits, odontogenic keratocysts of the jaw, multiple BCCs
G36	6/F	Deletion of the whole *PTCH1* gene			Bifid ribs, kyphoscoliosis, macrocephaly, frontal bossing, hypertelorism
G72	36/F	Frameshift	c.272delG c.274delT^*2^	p.G91VfsX26 p.C92VfsX25	Odontogenic keratocysts of the jaw, palmar pits, calcification of falx cerebri, stomach cancer

*1: Age: years of age when skin sample was taken, M: male, F: female.

*2: Patient “G72” had a germline mutation, c.272delG, and a low prevalence of somatic mutation, c.274delT, that was derived from the allele with a germline mutation, but not from the wild-type allele [25].

### Characterization of Gln-iPSCs

The proliferative capacity of three Gln-iPSC clones (G11, G36, G72) was measured and compared with that of Edom22-iPSCs (Figure 2B). No significant differences in proliferation rates were detected between the Gln-iPSC clones and Edom22-iPSCs.

### Teratoma formation

**Figure 3:**
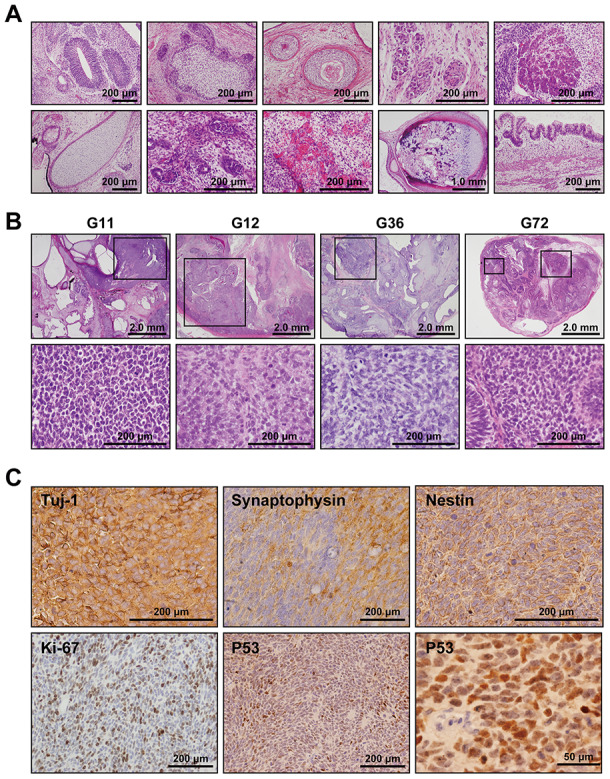
**Medulloblastoma in Gln iPSC-teratoma.** (**A**) Histology of teratoma generated by Gln-iPSCs. Upper panels from left to right: ectodermal glia and neuroepithelium; epidermis with hair follicles; epidermis with keratinization; ganglia; hepatocytes. Lower panels from left to right: cartilage; glomerulus-like structure; capillary vessels; bone and cartilage; intestinal epithelium. (**B**) Medulloblastomas were generated in the teratomas by Gln-iPSCs (G11, G12, G36, G72). Upper panels: low power view of the teratomas. Lower panels: high power view of medulloblastoma parts. Medulloblastomas were shown in the squares of the upper panels. (**C**) Immunohistochemical analysis of medulloblastoma using antibodies to Tuj-1, synaptophysin, nestin, Ki-67, and p53.

To address whether the Gln-iPSCs have the competence to differentiate into specific tissues, teratoma formation was tested by implantation of Gln-iPSCs in the subcutaneous tissue of immunodeficient Balb/c nu/nu mice. Gln-iPSCs produced teratomas within 6-12 weeks of implantation. Histological analysis of paraffin-embedded sections demonstrated that the three primary germ layers were generated as shown by the presence of ectodermal, mesodermal, and endodermal tissues in the teratoma (Figure 3A), implying that Gln-iPSCs have potential for multilineage differentiation in vivo. The areas of neuroepithelium, retina, and retinal pigmented epithelium were relatively large compared with those of other mesodermal and endodermal components.

### Medulloblastoma formation

**Figure 4:**
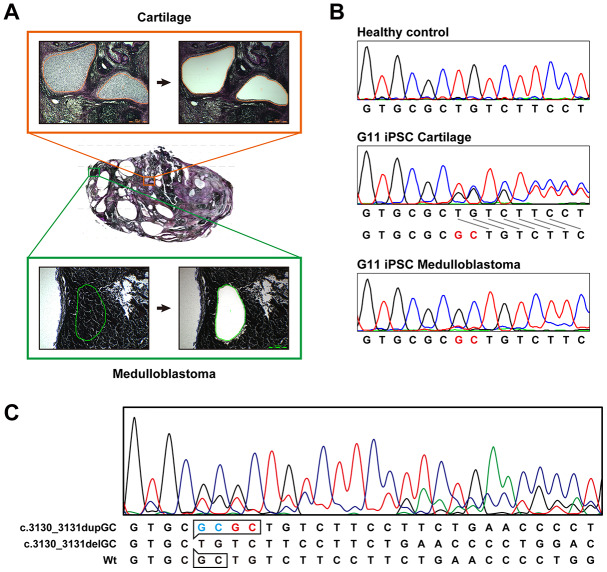
**Sequence analysis of the PTCH1 gene in medulloblastoma.** (**A**) Microdissection of medulloblastoma and cartilage. Genomic DNA was isolated from the microdissected medulloblastoma and applied to direct sequence analysis. Genomic DNA was also isolated from the cartilage for comparison. (**B**) Direct sequence analysis of genomic DNA from unaffected donor (control), G11-iPSC cartilage and G11-iPSC medulloblastoma. G11-iPSC cartilage had a duplication of “GC” (shown in red), and G11-iPSC medulloblastoma showed LOH of the *PTCH1* gene. (**C**) Direct sequence analysis of genomic DNA from G12-iPSC medulloblastoma. Sequences at the top and middle represent a germline mutation (c.3030_3031dupGC) and a somatic secondary mutation (c.3030_3031delGC), respectively. The minor wild-type sequence pattern (at the bottom) indicates contaminating non-medulloblastoma tissue.

Medulloblastoma formed in all the teratoma generated by all Gln-iPSC clones from the 4 different patients (Figure 3B). The diagnosis of medulloblastoma was confirmed by certified pathologists from two independent organizations. The tumor cells stained strongly positive for TUJ1, Synaptophysin, NESTIN, Ki67 and p53. For comparison, we used iPSCs of healthy and diseased donors. iPSCs such as PAE-iPSCs, UtE-iPSCs, AM-iPSCs, and 201B7 have been generated from placental, endometrial, amniotic, and fibroblastic cells. None of these iPSCs generated medulloblastomas in teratomas (0/584). Sequence analysis of the microdissected cartilage and medulloblastoma derived from G11 iPSCs revealed heterozygous mutations of the *PTCH1* gene and LOH of the *PTCH1* gene, respectively (Figure 4A, 4B). The medulloblastoma derived from G12 iPSCs contained c.3130_3131delGC instead of LOH in addition to c.3130_3131dupGC, suggesting that a small insertion or deletion as well as LOH can also cause medulloblastoma (Figure 4C).

## DISCUSSION

Human pluripotent stem cells deficient for a gene can be generated in two ways: Disruption of the gene in human ESCs or intact iPSCs by genetic manipulation with bacterial artificial chromosomes and derivation of disease-specific iPSCs from patients with germline mutations. Patient iPSCs serve as disease model cells for clarification of pathogenic mechanisms and for screening of novel compounds to treat the disease [10–12]. In this study, we generated iPSCs from fibroblasts of human Gorlin syndrome patients. Despite comparable proliferative activity, Gln-iPSCs consistently developed medulloblastoma, i.e. Gorlin syndrome-associated tumor, with secondary somatic mutations of the *PTCH1* gene.

Hereditary cancer syndrome-specific iPSCs have been analyzed in context of tumorigenesis in Li-Fraumeni syndrome and hereditary breast-ovarian cancer syndrome [14, 15]. These two cancer-prone genetic disorder-derived iPSCs exhibit disease-specific phenotypes, but do not develop cancers *in vivo*. In our study, however, medulloblastoma was generated in the teratomas derived from Gln-iPSCs established from four Gorlin individuals. Of these medulloblastomas, one exhibited somatic LOH at the *PTCH1* locus and another carried a somatic frameshift mutation in *PTCH1*. Since these somatic changes were not detectable before implantation, it is likely that they occurred during teratoma formation. Somatic loss of the *PTCH1* wild-type allele has been demonstrated in the sonic hedgehog subgroup of medulloblastomas (MB_SHH_) carrying germline *PTCH1* mutations [16]. Most of the MB_SHH_ lost the wild-type allele via LOH, and the others via single nucleotide variation, including insertion or deletion. Therefore, our study using Gln-iPSC recapitulates the development of medulloblastoma in patients with Gorlin syndrome.

Sonic hedgehog signaling in neural differentiation of human pluripotent stem cells may be related to the enlarged neuroectodermal component observed in the Gln-iPSC teratoma and medulloblastoma formation [17–19]. Mouse models of medulloblastoma, the most common pediatric brain tumor, have been generated. Ptc+/- mice on a B6D2F1 background develop medulloblastoma at a rate of 14%, and exhibit similar histology and anatomical location to human medulloblastoma [4]. Therefore, mice with genetically engineered *ptch1* haploinsufficiency are informative models in which to study Gorlin syndrome and medulloblastoma development. In contrast, medulloblastoma models using human cells have yet to be established. While all teratomas described in this study contained medulloblastomas, medulloblastoma develops in only 1-4% of the patients with Gorlin syndrome at an early ages [20]. The difference in frequency may be attributed to other factors such as cellular reprogramming in addition to hedgehog signaling. Formation of BCC in Gln-iPSC-derived teratoma was also expected because both sporadic and familial BCCs are often accompanied by LOH of the *PTCH1* gene as is the cases of medulloblastoma [21]. In contrast to medulloblastoma, BCC is basically a late onset tumor and its frequency in Japanese patients over 20 years of age barely exceeds 50% [20]. This late onset of BCC is possibly related with lack of BCC in the Gln-iPSC teratoma. An additional secondary mutation other than *PTCH1* LOH may be required for generation of BCC.

Alternatively, Ptc+/- mice on a mixed C57BL/6 DBA/2 background develop BCC only after ultraviolet and ionizing radiation [22]. Since the Gln-iPSC teratoma formed in the subcutaneous tissue was unexposed to chemical mutagens or such irradiation, the lack of BCC may be attributed to the controlled environment of the Gln-iPSC teratoma.

In conclusion, Gln-iPSCs may be a good model for medulloblastoma formation in Gorlin syndrome. These cells may also be useful for identification of biomarkers for medulloblastoma and for drug screening to identify treatments for this type of tumor. Moreover, Gln-iPSCs may serve as a good model to elucidate the mechanism of secondary somatic *PTCH1* mutations occurring in Gorlin syndrome-associated tumors.

## MATERIALS AND METHODS

### Human cells

Cells were obtained from four patients diagnosed with Gorlin syndrome carrying a confirmed *PTCH1* mutation at the time of surgery (Table 2) [23–27]. Fibroblasts were grown from non-tumor tissues. Cells were cultured in 100-mm dishes (Becton Dickinson). All cultures were maintained at 37°C in a humidified atmosphere containing 95% air and 5% CO_2_. When the cultures reached subconfluence, the cells were harvested with a Trypsin-EDTA solution (cat# 23315, IBL CO., Ltd, Gunma, Japan), and re-plated at a density of 5 x 10^5^ cells in a 100-mm dish. Medium changes were carried out twice a week thereafter. Edom22-iPSCs (Edom22iPS#S31) [9] and human embryonic stem cells (SEES-5, SEES-6 and SEES-7) were used as controls for Gln-iPSCs [28].

### Generation of iPSCs

iPSCs were generated according to the method supplied with the CytoTune-iPS 2.0 Sendai Reprogramming Kit (MBL). We employed the method for the iPSC generation because of its epigenetic effect [29]. Fibroblasts were seeded at 4.0 × 10^5^ cells per well in a 6-well plate 24 h before infection. Sendai viruses expressing human transcription factors *OCT3/4*, *SOX2*, *KLF4*, and *c-MYC* were mixed in fibroblast medium to infect fibroblasts according to the manufacturer’s instructions. Six days after transfection, the transduced cells were detached using Trypsin/EDTA solution (Wako) and passaged onto irradiated mouse embryonic fibroblast feeder cells in fibroblast medium. On the next day, the medium was exchanged with human iPSC medium. Human iPSC medium contained KO-DMEM, KSR, GlutaMAX, NEAA, 2-Mercaptoethanol, penicillin/streptomycin, sodium pyruvate and bFGF (all from Invitrogen). From the next day, the medium was changed every day and the culture dishes were monitored for the emergence of iPSC colonies. When colonies were ready for transfer, they were picked up and expanded. Elimination of Sendai virus was confirmed by RT-PCR. Cells just after infection served a positive control. Sequences of the primers set are: forward primer, 5’-AGA CCC TAA GAG GAC GAA GA-3’; reverse primer, 5’-ACT CCC GSG GCG TAA CTC CGS AGT G-3’.

### iPSC culture

Human iPSCs were cultured onto a feeder layer of freshly plated gamma-irradiated mouse embryonic fibroblasts, isolated from ICR embryos at 12.5 gestations and passages 2 times before irradiation (30 Gy), in the iPSC media or in a feeder-free condition in StemFit AK02N (AJINOMOTO) in 6-cm dishes coated with 0.5 μg/cm² vitronectin (Life Technologies). The cells were expanded using glass capillaries manually or passaging with CTK solution (ReproCELL). When passaging the cells to feeder free plates, the cells were dissociated into single cells by treatment with using 0.5× TrypLE Select (Life Technologies) (1× TrypLE Select diluted 1:1 with 0.5 mM EDTA/PBS(−)) and re-plated at a density of 6 cm dishes with StemFit media with 10 μM Y-27632 (Wako).

For EB formation, iPSC colonies were dissociated into single cells with accutase (Thermo Scientific, MA, USA) and then passaged into the low cell-adhesion 96 well plate dishes at a density of 10,000 cells/well in the iPSC medium without bFGF, and supplemented with ROCK inhibitor [28]. After confirming EB formation on day 7, the EBs were harvested and passage to dishes coated with Basement Membrane Matrix (354234, BD Biosciences). Thereafter the EBs were maintained for 14 days and changed the iPSC medium without bFGF every other day.

### Quantitative RT-PCR

Total RNA was isolated from cells using the RNeasy Plus Mini Kit (QIAGEN). cDNA was synthesized from 1 mg of total RNA using Superscript III reverse transcriptase (Invitrogen) with random hexamers according to the manufacturer’s instructions. Template cDNA was PCR-amplified with gene-specific primer sets (Supplementary Table 1). RNA was extracted from cells using the RNeasy Plus Mini kit (QIAGEN). An aliquot of total RNA was reverse transcribed using an oligo (dT) primer. For the thermal cycle reactions, the cDNA template was amplified (ABI PRISM 7900HT Sequence Detection System) with gene-specific primer sets using the Platinum Quantitative PCR SuperMix-UDG with ROX (11743-100, Invitrogen) under the following reaction conditions: 40 cycles of PCR (95°C for 15 s and 60°C for 1 min) after an initial denaturation (95°C for 2 min). Fluorescence was monitored during every PCR cycle at the annealing step. The authenticity and size of the PCR products were confirmed using a melting curve analysis (using software provided by Applied Biosystems) and a gel analysis. mRNA levels were normalized using GAPDH as a housekeeping gene.

### Immunocytochemical analysis

Cells were fixed with 4% paraformaldehyde in PBS for 10 min at 4°C. After washing twice with PBS and treatment with 0.2% tritonX-100 in PBS for 10 min at 4°C, cells were pre-incubated with blocking buffer (Protein Block Serum Free solution, DAKO) for 30 min at room temperature, and then reacted with primary antibodies in 1% BSA in PBS for overnight at 4°C. Following washing with PBS, cells were incubated with secondary antibodies; anti-rabbit or anti-mouse IgG conjugated with Alexa 488 or 546 (15300) (Invitrogen) in 1% BSA in PBS for 1 h at room temperature. Then, the cells were counterstained with DAPI and mounted.

### Immunohistochemistry

Immunohistochemistry was performed as previously described [30]. Paraffin sections were deparaffinized, dehydrated, and heated in Histofine Simple Stain MAX PO (MULTI) (Nichirei, Japan) for 20 min. After washing with distilled water, samples were placed in 1% hydrogen peroxide/methanol for 15 min to block endogenous peroxidase. Then, samples were incubated with blocking buffer (Protein Block Serum Free solution, DAKO) for 10 min at room temperature, the sections were then incubated at room temperature for 60 min in primary antibodies diluted with antibody diluent (Dako). The following primary antibodies against the antigens were used: Tuj-1 (1:300, Promega), Synaptophysin (1:400, DAKO), Nestin (1:200, Sigma-Aldrich), Ki-67 (1:100, Abcam), p53 (DAKO, 1:50). Then, they were washed three times with 0.01 M Tris buffered saline (TBS) solution (pH 7.4) and incubated with goat anti-mouse or anti-rabbit immunoglobulin labeled with dextran molecules and horseradish peroxidase (EnVision, Dako) at room temperature for 30 min. After washing with TBS, they were incubated in 3,3’-diaminobenzidin in substrate-chromogen solution (Dako) for 5-10 min. Negative controls were performed by omitting the primary antibody. The sections were counterstained with hematoxylin.

### Karyotypic analysis

Karyotypic analysis was contracted out at Nihon Gene Research Laboratories Inc. (Sendai, Japan). Metaphase spreads were prepared from cells treated with 100 ng/mL of Colcemid (Karyo Max, Gibco Co. BRL) for 6 h. The cells were fixed with methanol: glacial acetic acid (2:5) three times, and dropped onto glass slides (Nihon Gene Research Laboratories Inc.). Chromosome spreads were Giemsa banded and photographed. A minimum of 10 metaphase spreads were analyzed for each sample and karyotyped using a chromosome imaging analyzer system (Applied Spectral Imaging, Carlsbad, CA).

### Short tandem repeat analysis

Short tandem repeat analysis was contracted out to BEX Inc. (Tokyo, Japan) and the PowerPlex® 16 System(Promega) was employed. One primer specific for D3S1358, TH01, D21S11, D18S51, and Penta E was labeled with fluorescein (FL); one primer specific for D5S818, D13S317, D7S820, D16S539, CSF1PO, and Penta D was labeled with 6-carboxy-4′,5′-dichloro-2′,7′-dimethoxy-fluorescein (JOE); and one primer specific for Amelogenin, vWA, D8S1179, TPOX, and FGA was labeled with carboxy-tetramethylrhodamine (TMR). Genotyping of cell lines was analyzed by co-amplification of all sixteen loci and three-color detection.

### Teratoma formation

Gln-iPSCs were harvested by accutase treatment, collected into tubes, and centrifuged. The same volume of Basement Membrane Matrix (354234, BD Biosciences) was added to the cell suspension. The cells (>5.0×10^6^) were subcutaneously inoculated into immunodeficient mice (BALB/cAJcl-nu/nu, CREA, Tokyo, Japan). After 6 to 12 weeks, the resulting tumors were dissected and fixed with formalin. Paraffin-embedded tissue was sliced and stained with hematoxylin and eosin (HE). The operation protocols were accepted by the Laboratory Animal Care and the Use Committee of the National Research Institute for Child and Health Development, Tokyo. For comparison, we used iPSCs of healthy and diseased donors. iPSCs such as PAE-iPSCs, UtE-iPSCs, AM-iPSCs, and 201B7 have been generated from placental, endometrial, amniotic, and fibroblastic cells [8, 9, 31–34].

### Laser microdissection

Paraffin sections were cut as 10-micrometer sections onto PEN-membrane slides (Leica Microsystems, Wetzlar, Germany). Tissue sections were stained with hematoxylin. After drying the tissue, target area was extracted by use of a Leica LMD6500 (Leica).

### Mutational analysis

DNA was extracted using a DNeasy Blood & Tissue Kit (QIAGEN), QIAamp DNA mini kit (QIAGEN) or QIAamp DNA blood midi kit (QIAGEN). Genomic DNA was PCR-amplified with specific primer sets for the *PTCH1* gene (Supplementary Table 2). Amplified products were gel-purified using a QIAEX II gel extraction kit (QIAGEN) and cycle sequenced with a BigDye Terminator v3.1 Cycle Sequencing Kit (Applied Biosystems) in both directions. The sequence was analyzed on a 3130 Genetic Analyzer (Applied Biosystems). For some analyses, PCR products were subcloned into the pGEM-T Easy vector (Promega) and the inserts were sequenced.

### Ethical statement

Human cells in this study were obtained in full compliance with the Ethical Guidelines for Clinical Studies (Ministry of Health, Labor, and Welfare). The experimental procedure was approved by the Institutional Review Board (IRB) at National Center for Child Health and Development, Kitasato University and Chiba University Graduate School of Medicine.

## Supplementary Material

Supplementary Tables
