# Supplementary material for: Gorlin syndrome-induced pluripotent stem cells form medulloblastoma with loss of heterozygosity in PTCH1
Source: Aging (Albany NY). 2020 May 21;12(10):9935–47. doi: 10.18632/aging.103258 (PMC7288908; doi:10.18632/aging.103258)
Supplement: Supplementary Tables [file aging-12-103258-s001..pdf]

SUPPLEMENTARY TABLES

Supplementary Table 1. Primer sets for the pluripotency-associated genes.

	Forward (5'→3')	Reverse (5'→3')
OCT4/3	TGTACTCCTCGGTCCCTTTC	TCCAGGTTTTCTTTCCCTAGC
NANOG	CAGTCTGGACACTGGCTGAA	CTCGCTGATTAGGCTCCAAC
SOX2	ATGGGTTCGGTGGTCAAGT	GGAGGAAGAGGTAACCACAGG
DNMT3B	GGAAATTAGAATCAAGGAAATACGA	AATTTGTCTTGAGGCGCTTG
TERT	GGAGCAAGTTGCAAAGCATTG	TCCCACGACGTAGTCCATGTT
GAPDH	TGTTGCCATCAATGACCCCTT	CTCCACGACGTACTCAGCG

Supplementary Table 2. Primer sets to detect mutations of the PTCH1 gene.

	Forward (5'→3')	Reverse (5'→3')
G11(G12)	AACTGTGATGCTCTTCTACCCTGG	TCTTTCTGCAGCCGGGAAGTTTT
G36	CAACACCCAATTCTGGATAC	AAATCAGAGCCTGCATTCGC
G72	CACTCCTCCCTTCTGCTTCG	TCTGCCACGTATCTGCTCAC
